# Number of parity is associated with low-grade albuminuria in middle-aged and elderly Chinese women

**DOI:** 10.1186/s12905-019-0814-2

**Published:** 2019-10-07

**Authors:** Kan Sun, Diaozhu Lin, Feng Qiling, Feng Li, Yiqin Qi, Wanting Feng, Meng Ren, Li Yan, Dan Liu

**Affiliations:** Department of Endocrinology, Sun Yat-sen Memorial Hospital, Sun Yat-sen University, No.107 Yanjiang West Road, Guangzhou, 510120 People’s Republic of China

**Keywords:** Parity, Low-grade albuminuria, Cardiovascular diseases, Population-based study

## Abstract

**Background:**

Women with a higher number of pregnancies have a higher risk of developing cardiovascular diseases. Subtle fluctuations in albumin excretion could be related to pathophysiologic changes in the vascular system. We aimed to investigate the possible association of parity with low-grade albuminuria.

**Methods:**

We conducted a community-based study in 6495 women aged 40 years or older. Low-grade albuminuria was defined according to the highest quartile of urine albumin-to-creatinine ratio in participants free of micro- or macro-albuminuria.

**Results:**

Parous women with a higher number of pregnancies had increased age, body mass index (BMI), waist circumference (WC), systolic blood pressure (SBP), fasting plasma glucose (FPG), and fasting insulin, as well as decreased high-density lipoprotein cholesterol (HDL-C), estimated glomerular filtration rate (eGFR) levels, and proportion of menopause. The prevalence of low-grade albuminuria in parous women gradually increased with parity number. Compared with women with one childbirth, those with more than two childbirths were independently associated with a higher prevalent low-grade albuminuria (odds ratios [ORs] 1.41, 95% confidence interval [CI], 1.09–1.81) after multiple adjustments. In subgroup analysis after multiple adjustments, significant relation between parity number and prevalent low-grade albuminuria was detected in subjects age 55 years or older.

**Conclusion:**

Number of parity is associated with prevalent low-grade albuminuria in middle-aged and elderly Chinese women without micro- or macro-albuminuria.

## Background

Pregnancy related cardiometabolic changes could influence the health of women in later life. It is reported that an increasing number of pregnancies is associated with risk of cardiovascular diseases in women [1, 2]. Lawlor et al. [3] found that each additional child could increase the odds of coronary heart disease by 30% for women with at least two children. Data from the Trabzon Hypertension Study have shown a linear association between parity and the prevalent hypertension [4]. Moreover, Elisa et al. [5] demonstrated that parity is independently associated with early hypertension during menopausal transition. A recent meta-analysis has also suggested that elevated number of offspring in women is linearly associated with the risk of type 2 diabetes, particularly in those with multi-parity [6].

Moderately increased albuminuria reflects vascular endothelial dysfunction in the kidney. The well-known cut-off point of microalbuminuria is defined as a spot urine albumin-to-creatinine ratio (ACR) ≥ 30 mg/g [7, 8]. Albuminuria exceeding the upper limit is associated with increased risk of cardiovascular disease [9]. However, recent findings from prospective studies have suggested that low-grade albuminuria (ACR<30 mg/g), which was previously considered to be in the normal range, is related to an increased risk of cardiovascular morbidity and mortality in the general population [10, 11]. A prior study indicated that subclinical abnormalities of albuminuria could be related to elevated blood pressure in individuals without hypertension, which suggests that low-grade albuminuria could help to identify individuals most likely to progress to hypertension stages [12]. The above argument indicates the underlying biologic complexity of albumin excretion, in which subtle fluctuations in albumin excretion could be the manifestation of pathophysiology changes in the vascular system.

We assumed that number of offspring may have an effect on the early stage of albuminuria. However, epidemiological data focusing on the relationship between parity and albuminuria were surprisingly lacking. Therefore, the present study aimed to examine the association of parity degree with low-grade albuminuria in a general population who were free of micro- or macro-albuminuria.

## Methods

### Participants

We conducted a cross-sectional study in one community in Guangzhou, China, from June to November 2011. Further information about the survey, including study design and protocols, has been described previously [13–15]. In total, 9916 subjects signed the informed consent and enrolled into the survey. Firstly, men (*n* = 2854) were excluded from the study. After this, subjects who failed to provide information (ACR: *n* = 106; serum creatinine: *n* = 10) were also excluded. Of the remaining 6946 individuals, 451 subjects with ACR ≥ 30 mg/g were then excluded from the present analysis. Thus, a total of 6495 eligible individuals were included in the final data analyses.

### Data collection

We used standardized approaches to collect information on lifestyle and family characteristics. Information on reproductive history and personal socioeconomic information was self-reported. Women were asked to recall their number of pregnancies and parity. The International Physical Activity Questionnaire (IPAQ) was used as a comparable measure to estimate the frequency and duration of habitual physical activity [16]. Separate metabolic equivalent hours per week (MET-h/week) were calculated to evaluate the level of physical activity. Education levels were categorized as less than middle school, middle school graduate, and high school graduate or higher. Prior history of cardiovascular diseases, including previous coronary heart disease, myocardial infarction, stroke, and peripheral arterial disease, were collected in the baseline survey.

Body mass index (BMI) was calculated as weight in kilograms divided by height in meters squared (kg/m^2^). Waist circumferences (WC) was measured at the umbilical level with participants in the standing position. Obesity was defined as a BMI equal to or greater than 28, and overweight was defined as a BMI equal to or greater than 24 and less than 28 [17]. Overnight fasting blood samples of at least 10 h were collected for laboratory tests. Testing of triglycerides (TG), total cholesterol (TC), high-density lipoprotein cholesterol (HDL-C), low-density lipoprotein cholesterol (LDL-C), fasting plasma glucose (FPG), fasting insulin, and γ-glutamyltransferase (γ-GGT) was done using an autoanalyzer (Beckman CX-7 Biochemical Autoanalyzer, Brea, CA, USA). The Modification of Diet in Renal Disease (MDRD) study equation was used to calculate estimated glomerular filtration rate (eGFR) expressed in mL/min per 1.73 m^2^ using a formula of eGFR = 175 × [serum creatinine × 0.011]^-1.234^ × [age]^-0.179^ × [0.79 if female], where serum creatinine was expressed as μmol/L [18]. Hypertension was estimated using the Seventh Report of the Joint National Committee [19]. Diabetes was diagnosed according to the World Health Organization 1999 diagnostic criteria [20].

### Definition of low-grade albuminuria

Abnormalities in albumin excretion were defined according to the guidelines of the American Diabetes Association’s Standards of Medical Care [21]. The first morning spot urine samples were collected for testing the ACR. Urine albumin and creatinine were measured by chemiluminescence immunoassay (Siemens Immulite 2000, United States) and the Jaffe’s kinetic method (Biobase-Crystal, Jinan, China) on the automatic analyzer, respectively. The ACR was estimated by dividing the urinary albumin concentrations by the urinary creatinine concentrations and was expressed in mg/g. Increased urinary albumin excretion (micro- or macro-albuminuria) was defined as the ACR ranges ≥30 mg/g. Low-grade albuminuria was defined according to the highest quartile of ACR (≥ 11.54 mg/g in the study) in subjects without increased urinary albumin excretion.

### Statistical analysis

All statistical tests were two-sided, and a *P*-value < 0.05 was considered statistically significant. We performed all statistical analyses with SAS version 9.3 (SAS Institute Inc., Cary, NC, USA).

Continuous variables were presented as means ± standard deviation (SD) except for skewed variables, which were presented as medians (interquartile ranges). Categorical variables were expressed as numbers (proportions). On account of a non-normal distribution, FPG, TG, γ-GGT, and MET-h/week were logarithmically transformed before analysis. One-way ANOVA was used to test differences among groups and post hoc comparisons were performed by using Bonferroni correction. Comparisons between categorical variables were performed with the χ^2^ test. Pearson’s correlations were performed to test the correlations between risk factors for albuminuria and ACR. To identify potential confounding factors of the association between parity and ACR, variables significant at *P* <  0.20 in Pearson’s correlations were put into the multivariate stepwise linear regression models. The unadjusted and multivariate-adjusted logistic regression analyses were used to assess prevalent low-grade albuminuria in relation to degree of parity. Covariates significant in the stepwise linear regression were put into multivariate logistic regression analysis. Model 1 was unadjusted. Model 2 was adjusted for age. Model 3 was further adjusted for SBP, TG, HDL-C, FPG, eGFR, and physical activity levels. Model 4 was adjusted for age, SBP, TG, HDL-C, FPG, eGFR, physical activity levels, education levels, and prior history of CVD. Odds ratios (ORs) and the corresponding 95% confidence intervals (95% CI) were calculated. Relationship of parity numbers (per one live birth increase) with low-grade albuminuria was examined in subgroups stratified by age (≥ 55/< 55 years), degree of obesity (normal/overweight/obese), hypertension (yes/no), diabetes (yes/no), and eGFR levels (≥ 90; 60–89; < 60 ml/min. Per 1.73 m^2^). Tests for interaction were estimated by simultaneously including each strata factor, parity degree, and interaction term (strata factor multiplied by parity degree) in the multivariate-adjusted model.

## Results

### Basic characteristics of the study population

The mean age among the 6495 enrolled women in the study was 55.0 ± 7.6 years. In total, 65.2% (4236) of the in this study women were one childbirth and 12.6% (820) of the women were nulliparous. Compared to subjects without low-grade albuminuria, those with low-grade albuminuria were older and had higher BMI, WC, SBP, DBP, TG, TC, FPG, fasting insulin, and γ-GGT (Table 1, all *P* <  0.05).

**Table 1:** General characteristics of the study population

	Participants without low-grade albuminuria	Participants with low-grade albuminuria	*P*-values
n (%)	4871 (75.0)	1624 (25.0)	
Urinary ACR (mg/g)	7.0 (5.4–8.8)	15.1 (13.0–19.4)	< 0.0001
Age (years)	54.7 ± 7.4	56.1 ± 8.1	< 0.0001
BMI (kg/m^2^)	23.4 ± 3.2	24.0 ± 3.9	< 0.0001
WC (cm)	79.8 ± 8.9	81.2 ± 9.5	< 0.0001
SBP (mmHg)	123.0 ± 15.4	128.1 ± 16.8	< 0.0001
DBP (mmHg)	73.6 ± 9.1	75.4 ± 10.2	< 0.0001
Current smoking [n (%)]	55 (1.2)	23 (1.5)	0.353
Current drinking [n (%)]	58 (1.2)	23 (1.5)	0.478
TG (mmol/L)	1.20 (0.88–1.69)	1.30 (0.92–1.91)	< 0.0001
TC (mmol/L)	5.23 ± 1.26	5.34 ± 1.27	0.0003
HDL-C (mmol/L)	1.38 ± 0.37	1.37 ± 0.36	0.223
LDL-C (mmol/L)	3.16 ± 0.97	3.20 ± 0.99	0.106
FPG (mmol/L)	5.36 (4.97–5.82)	5.45 (5.02–5.97)	< 0.0001
Fasting insulin (μIU/ml)	7.10 (5.30–9.80)	7.80 (5.50–10.80)	< 0.0001
γ-GGT (U/L)	17.0 (13.0–24.0)	19.0 (14.0–27.0)	< 0.0001
eGFR (ml/min per 1.73 m^2^)	104.3 ± 22.5	104.7 ± 24.2	0.590
Physical activity (MET-h/week)	22.0 (10.5–45.0)	21.0 (10.5–42.0)	0.092
Prior history of CVD [n (%)]	139 (2.9)	46 (2.8)	0.965
High school or higher education [n (%)]	2892 (61.4)	912 (58.4)	0.017
Spontaneous abortion [n (%)]	330 (6.8)	92 (5.7)	0.116
Menopause [n (%)]	1160 (25.2)	333 (21.7)	0.005

1. Data were means ± SD or medians (interquartile ranges) for skewed variables or numbers (proportions) for categorical variables; *P*-values were for the ANOVA or χ^2^ analyses between the two groups

2. *ACR* albumin to creatinine ratio, *BMI* body mass index, *WC* waist circumference, *SBP* systolic blood pressure, *DBP* diastolic blood pressure, *TG* triglycerides, *TC* total cholesterol, *HDL-C* high-density lipoprotein cholesterol, *LDL-C* low-density lipoprotein cholesterol, *FPG* fasting plasma glucose, *eGFR* estimated glomerular filtration rate, *γ-GGT* γ-glutamyltransferase, *CVD* cardiovascular diseases

Clinical and biochemical characteristics of the participants according to parity degree are shown in Table 2. Compared with nulliparous women, women who had just one live birth in their life were younger and had lower TC, LDL-C, and γ-GGT levels, and lower proportions of them were current smokers and current drinkers. Parous women with higher parity number had higher ACR, age, BMI, WC, SBP, FPG, fasting insulin, and prior history of CVD, as well as lower HDL-C, eGFR levels, proportion of menopause, and education levels.

**Table 2:** Characteristics of study population by number of parity category

	Number of Parity			
	0	1	2	≥ 3
n (%)	820 (12.6)	4236 (65.2)	981 (15.1)	458 (7.1)
Urinary ACR (mg/g)	8.0 (5.8–11.5)	8.0 (5.8–11.2)	8.6 (6.3–11.9) ^#, &^	9.3 (6.4–13.1) ^#, &^
Age (years)	54.3 ± 7.1^#^	53.4 ± 5.6 ^&^	58.1 ± 8.7 ^#, &^	65.3 ± 10.9 ^#, &^
BMI (kg/m^2^)	23.2 ± 3.2	23.4 ± 3.4	24.1 ± 3.2 ^#, &^	24.6 ± 3.8 ^#, &^
WC (cm)	79.3 ± 10.0	79.3 ± 8.6	82.3 ± 9.1 ^#, &^	84.8 ± 9.4 ^#, &^
SBP (mmHg)	123.6 ± 15.3	123.0 ± 15.6	127.0 ± 16.0 ^#, &^	131.8 ± 17.1 ^#, &^
DBP (mmHg)	74.1 ± 9.1	73.8 ± 9.4	74.6 ± 9.5	74.6 ± 9.7
Current smoking [n (%)]	21 (2.8) ^#^	37 (0.9) ^&^	11 (1.1) ^&^	9 (2.0) ^#^
Current drinking [n (%)]	14 (2.0) ^#^	45 (1.1) ^&^	16 (1.7)	6 (1.3)
TG (mmol/L)	1.25 (0.92–1.78) ^#^	1.18 (0.87–1.69) ^&^	1.28 (0.97–1.84) ^#^	1.43 (0.99–1.99) ^#, &^
TC (mmol/L)	5.43 ± 1.24 ^#^	5.24 ± 1.27 ^&^	5.25 ± 1.27 ^&^	5.22 ± 1.26 ^&^
HDL-C (mmol/L)	1.42 ± 0.36	1.39 ± 0.37	1.36 ± 0.35 ^&^	1.28 ± 0.35 ^#, &^
LDL-C (mmol/L)	3.26 ± 0.97 ^#^	3.15 ± 0.98 ^&^	3.16 ± 0.96	3.15 ± 0.95
FPG (mmol/L)	5.35 (4.94–5.82)	5.35 (4.95–5.80)	5.50 (5.07–6.00) ^#, &^	5.60 (5.13–6.09) ^#, &^
Fasting insulin (μIU/ml)	7.10 (5.10–9.85)	7.10 (5.20–9.70)	7.80 (5.80–10.8) ^#, &^	8.20 (5.90–11.1) ^#, &^
γ-GGT (U/L)	18.0 (14.0–26.0) ^#^	17.0 (13.0–24.0) ^&^	19.0 (14.0–27.0) ^#^	19.0 (14.0–26.0) ^#^
eGFR (ml/min per 1.73 m^2^)	103.2 ± 23.0 ^#^	106.0 ± 23.2 ^&^	102.3 ± 20.8 ^#^	96.9 ± 22.2 ^#, &^
Physical activity (MET-h/week)	10.5 (0.0–36.0)	24.8 (11.8–49.0)	28.0 (12.0–49.0)	21.0 (10.5–42.0) ^#^
Prior history of CVD [n (%)]	14 (1.7)	81 (1.9)	48 (4.9) ^#, &^	42 (9.7) ^#, &^
High school or higher education [n (%)]	463 (70.6)	2902 (69.2)	379 (39.2) ^#, &^	60 (13.3) ^#, &^
Spontaneous abortion [n (%)]	27 (3.3) ^#^	265 (6.3) ^&^	73 (7.4) ^&^	57 (12.5) ^#, &^
Menopause [n (%)]	139 (25.5)	1111 (26.6)	189 (19.5) ^#, &^	54 (11.8) ^#, &^

1. Data were means ± SD or medians (interquartile ranges) for skewed variables or numbers (proportions) for categorical variables; *P*-values were for the ANOVA or χ^2^ analyses across the groups

2. ^#^*P* <  0.05 compared with participants with one live births (parity number equal to the 1 group); ^&^ *P* <  0.05 compared with participants with no live birth (parity number equal to the 0 group)

3. *ACR* albumin to creatinine ratio, *BMI* body mass index, *WC* waist circumference, *SBP* systolic blood pressure, *DBP* diastolic blood pressure, *TG* triglycerides, *TC* total cholesterol, *HDL-C* high-density lipoprotein cholesterol, *LDL-C* low-density lipoprotein cholesterol, *FPG* fasting plasma glucose, *eGFR* estimated glomerular filtration rate, *γ-GGT* γ-glutamyltransferase

Pearson’ s correlation analyses revealed that age, BMI, WC, SBP, DBP, TG, HDL-C, FPG, fasting insulin, γ-GGT, eGFR, menopause proportion, history of CVD, and education levels were significantly correlated with ACR. After performing multivariate stepwise linear regression analysis, we found that age, SBP, TG, HDL-C, FPG, eGFR, and physical activity levels were independent determinants for ACR (all *P* <  0.05, Table 3).

**Table 3:** Pearson’s correlation and stepwise regression analysis of determinants of ACR

	r	*P* value	Standardized β	*P* value
Age (years)	0.11	< 0.0001	0.10	< 0.0001
BMI (kg/m^2^)	0.04	0.0009	–	–
WC (cm)	0.06	< 0.0001	–	–
SBP (mmHg)	0.15	< 0.0001	0.11	< 0.0001
DBP (mmHg)	0.09	< 0.0001	–	–
Current smoking [n (%)]	0.02	0.20	–	–
Current drinking [n (%)]	0.005	0.673	–	–
TG (mmol/L)	0.08	< 0.0001	0.05	0.0003
TC (mmol/L)	0.02	0.129	–	–
HDL-C (mmol/L)	−0.03	0.017	0.05	0.0017
LDL-C (mmol/L)	0.002	0.829	–	–
FPG (mmol/L)	0.10	< 0.0001	0.07	< 0.0001
Fasting insulin (μIU/ml)	0.05	0.0002	–	–
γ-GGT (U/L)	0.07	< 0.0001	–	–
eGFR (ml/min per 1.73 m^2^)	0.05	0.0002	0.11	< 0.0001
Physical activity (MET-h/week)	−0.02	0.104	−0.03	0.0390
Prior history of CVD [n (%)]	−0.03	0.037	–	–
High school or higher education [n (%)]	−0.05	< 0.0001	–	–
Spontaneous abortion [n (%)]	−0.005	0.664	–	–
Menopause [n (%)]	−0.06	< 0.0001	–	–

r, correlation coefficient; β, regression coefficient

### Parity degree in relation to low-grade albuminuria

The prevalence of low-grade albuminuria was 24.8, 23.5, 27.4, and 34.5% among subjects in parity number in the 0, 1, 2, and ≥ 3 groups, respectively. Strikingly, a significant increase of prevalent low-grade albuminuria was observed in parity number in the 2 and ≥ 3 groups when compared with parity number in the 1 group (*P* = 0.0009 and <  0.0001, respectively).

**Fig. 1:**
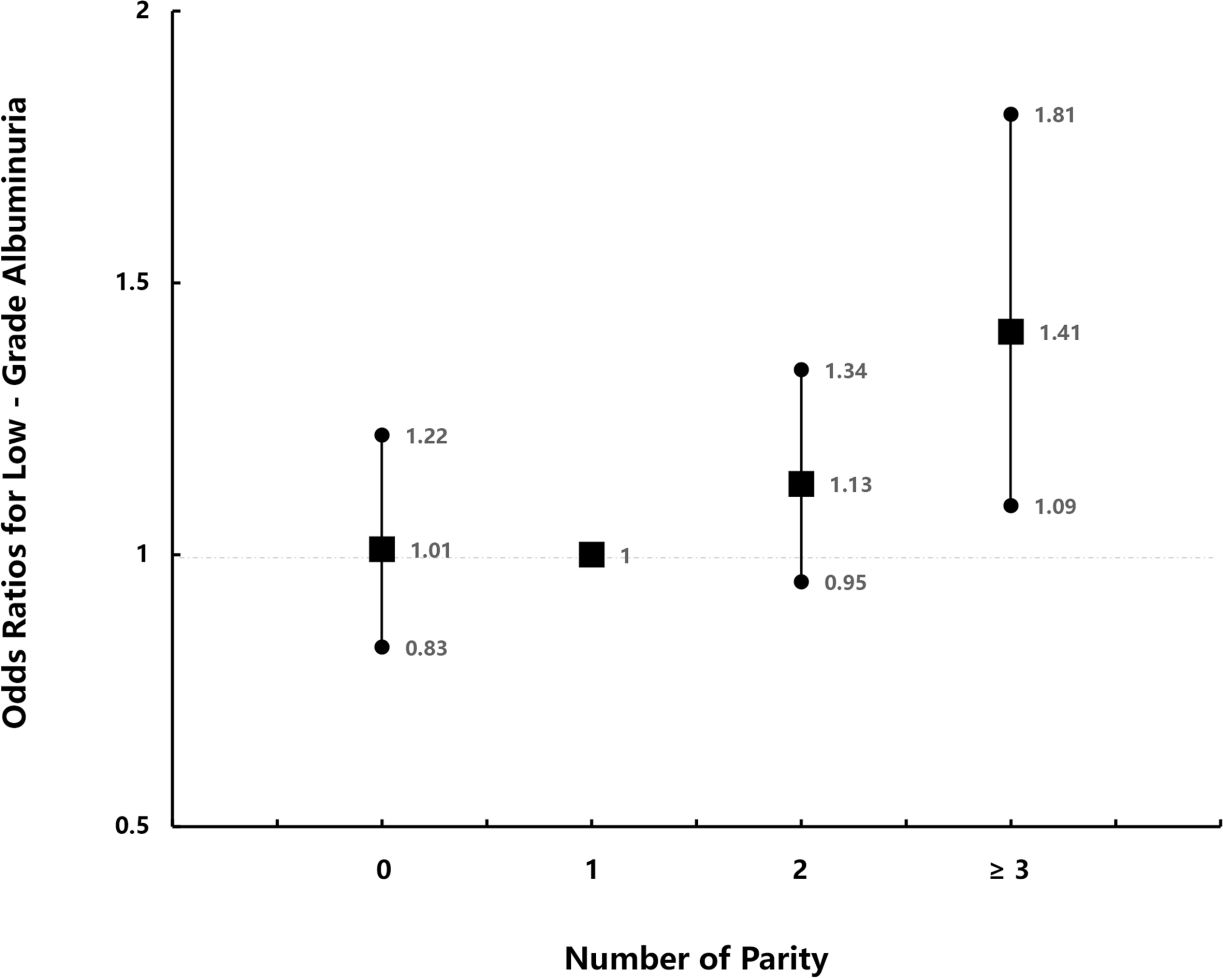
Multivariate logistic regression analyses of parity number with prevalent low-grade albuminuria

Compared with women with one childbirth (parity number = 1 group), univariate logistic regression analysis in Model 1 showed that subjects with two live births (parity number = 2 group) and with more than two live births (parity number ≥ 3 group), respectively, had a significant correlation with increased odds of prevalent low-grade albuminuria (Table 4). As shown in Fig. 1, in multivariate logistic regression analyses, subjects with more than two live births were independently associated with a greater prevalence of low-grade albuminuria (ORs 1.41, 95% CI, 1.09–1.81) when compared with women with one childbirth. However, in multivariate analyses, no significant difference in such associations was found when comparing women with one childbirth to nulliparous women (parity number = 0 group) or to those with two live births (parity number = 2 group).

**Table 4:** The risk of prevalent low-grade albuminuria according to elevated parity degree

	Number of Parity			
	0	1	2	≥ 3
Low-grade albuminuria				
Model 1	1.07 (0.90–1.28)	1	1.23 (1.05–1.44)	1.72 (1.40–2.11)
Model 2	1.06 (0.89–1.26)	1	1.14 (0.97–1.34)	1.39 (1.11–1.75)
Model 3	1.03 (0.86–1.23)	1	1.11 (0.94–1.31)	1.33 (1.06–1.69)
Model 4	1.01 (0.83–1.22)	1	1.13 (0.95–1.34)	1.41 (1.09–1.81)

Data are odds ratios (95% confidence interval). Participants without low-grade albuminuria are defined as 0 and with low-grade albuminuria as 1

Model 1 is unadjusted

Model 2 is adjusted for age

Model 3 is adjusted for age, SBP, TG, HDL-C, FPG, eGFR, and physical activity levels

Model 4 is adjusted for age, SBP, TG, HDL-C, FPG, eGFR, physical activity, education levels, and prior history of CVD

**Fig. 2:**
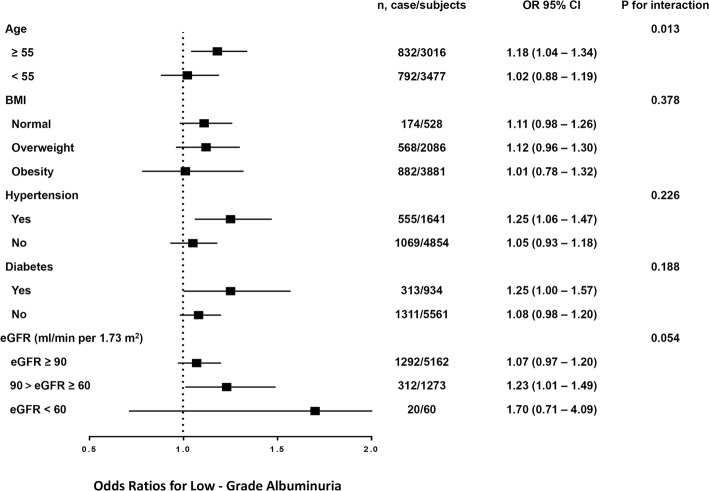
Risk of prevalent low-grade albuminuria with elevated parity degree in different subgroups

According to stratified analyses in Fig. 2, the associations between parity degree and low-grade albuminuria in multivariate analyses were not consistently the same, and significant difference of such relationship was detected in subjects age ≥ 55 years, those with hypertension, and those with 90>eGFR ≥60 ml/min*1.73 m^2^. Moreover, statistical significance of interaction term between parity degree and age stratification was also detected.

## Discussion

In this study of the Chinese population with ACR less than the current microalbuminuria threshold, we found that higher parity degree was significantly associated with increasing risk of prevalent low-grade albuminuria. The association remained after adjusting for conventional risk factors and intermediates. To our current knowledge, no previous studies have provided evidence that parity degree is independently associated with low-grade albuminuria.

Using creatinine-based equations in detecting subtle changes in renal filtration function has inherent insensitivity and limitations in the early stages of kidney damage. In the present study, a positive association between eGFR and albuminuria was found. It is possible that kidney damage of the subjects in the cohort was in the early stage, as the average eGFR levels were still in the normal range. We assumed that prodromal renal hyper-filtration and increased glomerular pressure in the early stage of chronic kidney disease could be the cause of increased urinary albumin in the present study. Actually, in all models of logistic regression analysis, parity degree was independently associated with a greater prevalence of low-grade albuminuria even after adjustment for eGFR. Generally, 30 mg/g of ACR is considered the cut-off point of increased urinary excretion of albumin and used to predict chronic kidney disease. Recent studies have declared that the average ACR level is actually much lower in the early stage of kidney disease [22, 23]. Low-grade albuminuria, even within the previously defined normal range, is associated with development and progression of cardiovascular disease, which has received great attention in recent years [24, 25].

The present study extended the results of previous studies by confirming the association between parity degree and increased risk of prevalent low-grade albuminuria [3, 6, 12, 26]. Pregnancy produces significant alterations in women’s bodies, which may lead to constant but not temporary influence on women’s health [6, 27]. In fact, a higher number of offspring is also associated with lower socioeconomic status and child-rearing-related lifestyle risk factors. The accumulative effect that women experience in their later life could promote weight gain, insulin resistance, and dyslipidemia, which are often cited as adverse risk factors for cardiovascular diseases [3, 28]. The test for interaction between age and parity was significant, supporting an age difference for the association. Moreover, when comparing women with one childbirth to nulliparous women or to those with two childbirths, no significant difference regarding the relationship between parity of low-grade albuminuria was detected. Such results were consistent with some of the previous studies; therefore, our findings suggest that parity degree may have an accumulation effect with albuminuria risk in this population, which may be diluted by low risk in women with relatively fewer births [28].

The study highlights the importance of paying clinical attention to early albuminuria in women with multi-parity. The present findings emphasize that increasing is associated with subtle fluctuations in albumin excretion, which may reflect in pathophysiologic changes in the microvascular system. Moreover, as micro- and macroalbuminuria are much more serious manifestation of renal injury, it is likely that there are more metabolic risk factors associated with micro- and macroalbuminuria, some of which might veil the effect of parity degree. However, the underlying factors remain unclear and need further exploration. Reported data of abnormal albuminuria as low-grade or micro- and macroalbuminuria together could attenuate the main findings of this study, so we excluded individuals with increased urinary albumin excretion from the cohort.

Some biological and socioeconomic mechanisms that reflect pregnancy-related physiological changes may account for the possible link between number of offspring and low-grade albuminuria. The increase in parity degree with increased exposure to arterial hypertension and anti-insulin hormones may represent a combination of short-term effects of parity on susceptible subjects who have gestational hypertension and diabetes, and long-term effects on the macro- and micro-vascular system who have arteriosclerotic cardiovascular disease and increased urinary albumin excretion [28–30]. Another possible interpretation is that a higher number of offspring is usually related to lifestyle and socioeconomic status change, which may have potential influence on the risk of later albuminuria [31, 32]. Actually, both the harmful and protective aspects of these factors may take part in albuminuria development; thus, based on our findings, we suggest that unhealthy lifestyle characteristics, such as cigarette smoking, excessive drinking, and poor dietary habit, be eliminated, especially in families with high parity degree.

There are several limitations to be considered. Firstly, the cross-sectional design was a limitation of this study, and no causal inference can be drawn. The prospective association of parity degree with incident low-grade albuminuria in other cohorts is needed to verify our findings. Moreover, we will aim to conduct longitudinal research to examine the association between parity and outcomes of cardiovascular diseases, after adjustment for albuminuria. Secondly, self-reported information on pregnancies was not accurate enough, as recall bias may have affected association of parity degree with low-grade albuminuria in the present study. More detailed and accurate information about the disorders during the pregnancies or abnormal obstetrical outcomes (e.g. preterm labor, pregnancy hypertension, fetal growth restriction) should be collected to strengthen the findings of the study. Thirdly, as mentioned in our previous publication, urinary albumin excretion was evaluated on a spot morning urine sample, which may not have accurately reflected the true level of albuminuria [33]. Actually, 24-h urine collection or three samples from three consecutive days would have provided more stable results for albumin excretion [34]. However, spot specimens for urinary ACR correlate well with those of 24-h collection and multiple urine samples, so urinary ACR assessment by spot samples could be a reliable alternative in epidemiological specimen collection [35, 36]. Fourthly, although we adjusted for a spectrum of covariates associated with ACR in the multivariate regression analyses, other potential mediators, such as social status, personal income levels, and family lifestyle factors, could potentially have been residually confounding and should have been adjusted in the present study. Despite the above limitations, the current study included a large community-based cohort of individuals and was the first to examine the association between parity degree and risk of prevalent low-grade albuminuria, both of which add to the strength of our findings.

## Conclusions

In conclusion, parity degree is independently associated with prevalence of low-grade albuminuria in middle-aged and elderly Chinese women. Our study is the first to emphasize the importance of paying clinical attention to early albuminuria in women with an increased number of offspring. Further studies with other ethnic groups and prospective designs are needed to verify our findings.

## Data Availability

The datasets used and/or analyzed during the current study are available from the corresponding author on reasonable request.

## Abbreviations

ACR: Albumin to creatinine ratio
BMI: Body mass index
CVD: Cardiovascular diseases
DBP: Diastolic blood pressure
eGFR: Estimated glomerular filtration rate
FPG: Fasting plasma glucose
HDL-C: High-density lipoprotein cholesterol
LDL-C: Low-density lipoprotein cholesterol
SBP: Systolic blood pressure
TC: Total cholesterol
TG: Triglycerides
WC: Waist circumference
γ-GGT: γ-glutamyltransferase
